# Correction: An eight-year follow-up on auditory outcomes after neonatal hearing screening

**DOI:** 10.1371/journal.pone.0308470

**Published:** 2024-08-02

**Authors:** Jolien J. G. Kleinhuis, Karin de Graaff-Korf, Henrica L. M. van Straaten, Paula van Dommelen, Michel R. Benard

The images for Figs 7 and 8 are incorrectly switched. The image that appears as Fig 7 should be Fig 8, and the image that appears as Fig 8 should be Fig 7. The figure captions appear in the correct order.

**Fig 7:**
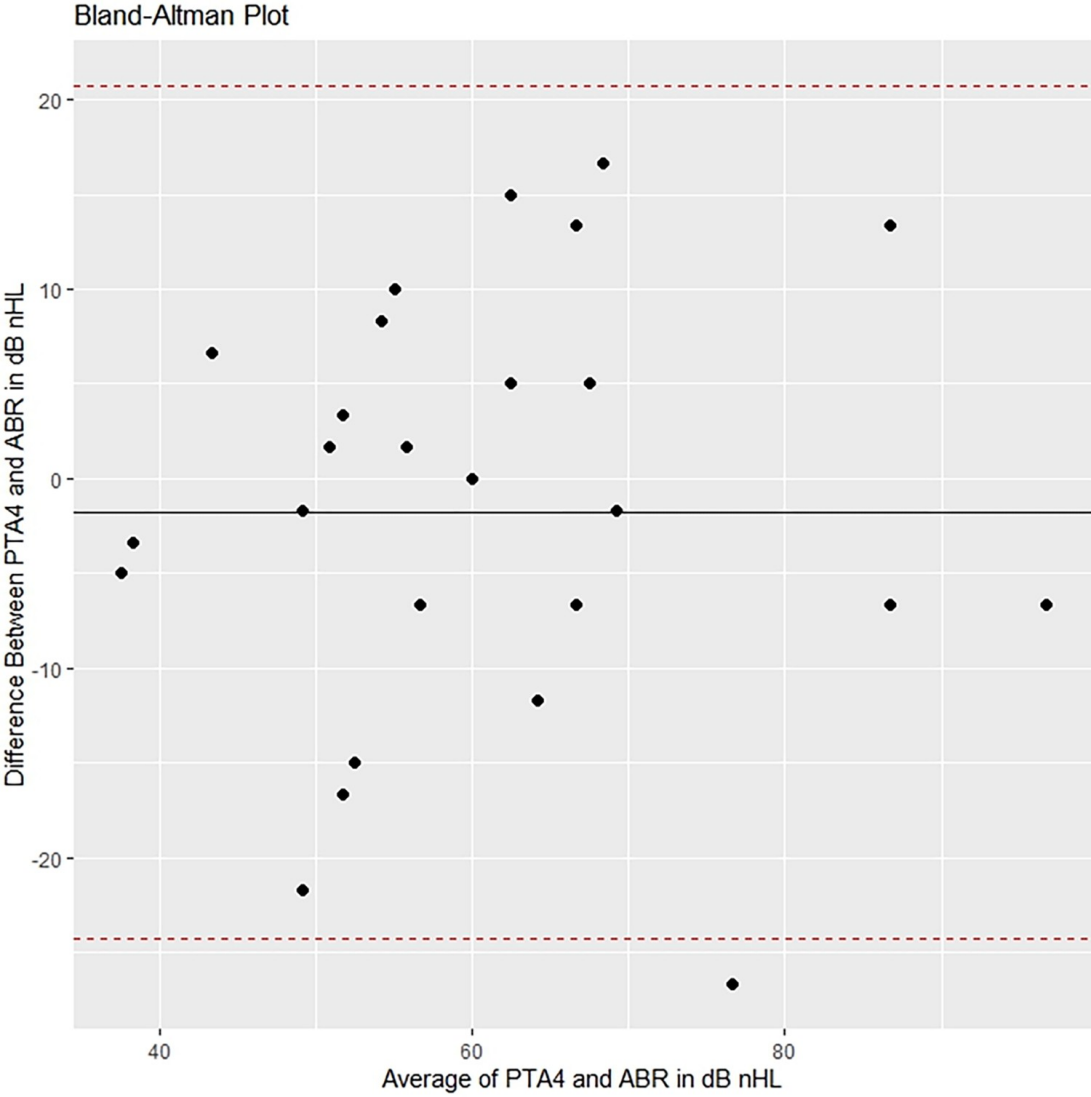
Bland-Altman plot comparing ABR with PTA at age 4.

**Fig 8:**
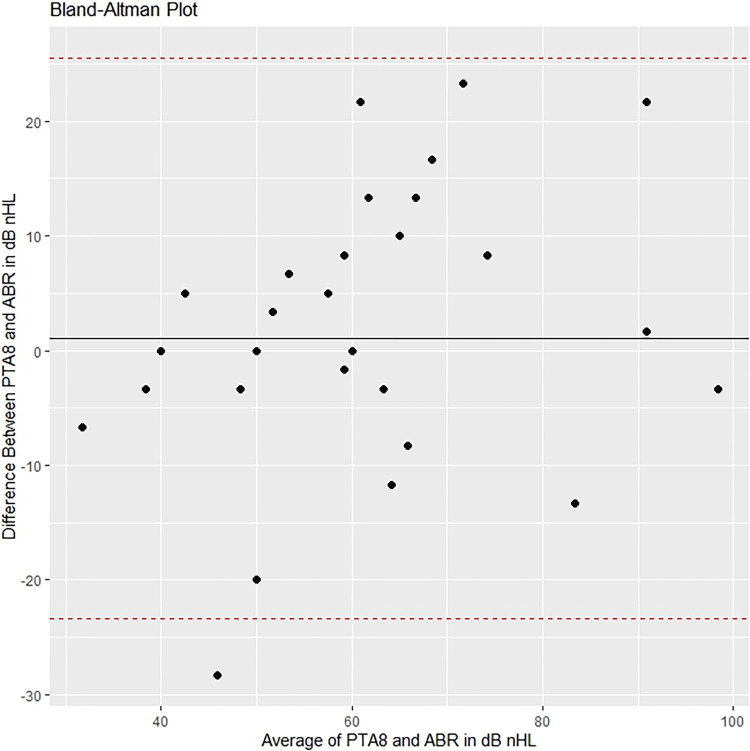
Bland-Altman plot comparing ABR with PTA at age 8.
