# Supplementary material for: An eight-year follow-up on auditory outcomes after neonatal hearing screening
Source: PLoS One. 2024 Feb 28;19(2):e0297363. doi: 10.1371/journal.pone.0297363 (PMC10901348; doi:10.1371/journal.pone.0297363)
Supplement: Attachment — Submitted filename: Response to Reviewers.docx [file pone.0297363.s001.docx]

Dear editor, dear reviewers,

We express sincere gratitude to the reviewers and the editor for their invaluable comments. We aspire that, with the implemented modifications and additions, we will once again be considered for publication. Below we provide a point by point answer to all comments received.

Kind regards,

Jolien Kleinhuis

Also on behalf of all the other authors:

Karin de Graaff, Paula van Dommelen, Irma van Straaten en Ruben Benard

Reviewer's Responses to Questions

Comments to the Author

1. Is the manuscript technically sound, and do the data support the conclusions?

The manuscript must describe a technically sound piece of scientific research with data that supports the conclusions. Experiments must have been conducted rigorously, with appropriate controls, replication, and sample sizes. The conclusions must be drawn appropriately based on the data presented.

Reviewer #1: Partly

Reviewer #2: Yes

**Answer:** *We are happy to read that Reviewer 2 believes that the manuscript is technically sound and that the results support the conclusions. Reviewer 1 provides some very good suggestions later in this document that we will take to heart, and we will address them later.*

________________________________________

2. Has the statistical analysis been performed appropriately and rigorously?

Reviewer #1: Yes

Reviewer #2: Yes

________________________________________

3. Have the authors made all data underlying the findings in their manuscript fully available?

The PLOS Data policy requires authors to make all data underlying the findings described in their manuscript fully available without restriction, with rare exception (please refer to the Data Availability Statement in the manuscript PDF file). The data should be provided as part of the manuscript or its supporting information, or deposited to a public repository. For example, in addition to summary statistics, the data points behind means, medians and variance measures should be available. If there are restrictions on publicly sharing data—e.g. participant privacy or use of data from a third party—those must be specified.

Reviewer #1: Yes

Reviewer #2: Yes

________________________________________

4. Is the manuscript presented in an intelligible fashion and written in standard English?

PLOS ONE does not copyedit accepted manuscripts, so the language in submitted articles must be clear, correct, and unambiguous. Any typographical or grammatical errors should be corrected at revision, so please note any specific errors here.

Reviewer #1: Yes

Reviewer #2: Yes

**Answer:** *We are very pleased that points 2 through 4 have been answered with 'yes.' We are happy to read that the effort we have invested in this manuscript have resulted in this - in our view - positive review*

________________________________________

5. Review Comments to the Author

Please use the space provided to explain your answers to the questions above. You may also include additional comments for the author, including concerns about dual publication, research ethics, or publication ethics. (Please upload your review as an attachment if it exceeds 20,000 characters)

Reviewer #1: Dear authors,

After carefully reviewing your manuscript, I would like to extend my congratulations on your work. Overall, the presented data seems interesting and appropriately collected and analyzed.

**Answer:** *Thank you for your congratulations; we share your opinion that the results are important for our field of work*

However, I believe that the main objectives described in the paper are not fully addressed. For instance, the study aims to evaluate the predictive value of the initial click ABR in healthy children regarding their perceptual auditory thresholds throughout their life up to the age of 8 years. Unfortunately, the amount of missing data is significant, and the sample size at 8 years is relatively low

**Answer:** *Thank you for clearly sharing your concerns about our article. As we read your comment, we realize that we may need to provide more explanation for both Figure 1 and Figure 4 in the manuscript. Firstly, regarding the total sample size, out of the 135 children, we were able to follow 106 children to completion, so 29 children were lost to follow-up. These children represent various types of hearing diagnoses. However, when we specifically consider children with permanent hearing losses, for instance, among the 40 children with bilateral hearing losses at eight years old, 30 were still included in our study. Given the lengthy duration of this study, which spans eight years, we believe that a 75% participation rate is high. Moreover, we believe that the data were representative after 8 years because the main reasons for no follow up were that follow up took place at another institute (n=2) or were referred for cochlear implants (n=8). We have now added the reasons for non-follow-up in our results section, to make it clear that there were no children who did not want to follow up. However, we agree that the numbers were relatively small within some subgroups, we therefore tried to provide as much insight into the data at the individual level, as with the Bland-Altman plots.*

Additionally, it is widely known that there is a relationship between electrophysiological and perceptual thresholds in newborns.

**Answer:** *In the literature, the relationship between ABR results and the hearing threshold in young children has certainly been demonstrated, as also discussed in our introduction and discussion sections. The strength of this study lies in demonstrating this relationship over a span of eight years. This has clinical relevance for clinics worldwide that utilize click stimuli for ABR testing.*

If a child's hearing remains unchanged over time, these thresholds will naturally remain equivalent. In such cases, it would be very interesting to have a report on the observed rate of progressive sensorineural hearing loss in your cohort.

**Answer:** *We completely agree with you; it is indeed interesting to study the evolution over time, not only in cases of stable hearing thresholds but especially in those with fluctuating thresholds. This is depicted in Figure 4 and also in the Bland-Altman plots.*

The second objective was to determine which perceptual tonal average (1,2, 4 kHz, or 2.4 kHz) best matches the electrophysiological threshold. In this regard, I believe it is essential to contrast the results of the assessments considering the profile of the auditory curve (ascending, descending, flat, among others).

**Answer:** *This is indeed a good point. To address this point we classified the shapes of all SNHL audiograms and correlated them with two distinct perceptual tonal averages. We believed this addition would enhance our work, and we sincerely appreciate your insightful suggestion. However, upon careful consideration, the results, in our judgment, do not contribute significantly to the overall findings. Despite the value we place on conducting this analysis, prompted by your remark, it appears impractical to include this aspect in the manuscript. Our approach involved the classification of audiogram shapes obtained at ages 4 and 8 as follows:Flat: Hearing loss is present at all frequencies, and the difference between frequencies is ≤ 5 dB, or the trendline is distinctly horizontal.*

- *Ascending: Hearing thresholds decrease with increasing frequency, or the trendline is ascending.*
- *Descending: Hearing thresholds increase with increasing frequency, or the trendline is descending.*
- *Cookie-Bite: Hearing thresholds at middle frequencies are worse than at lower and higher frequencies, or the trendline exhibits a cookie-bite shape."*

*The analyzed data is found in table 1, where the mean ABR threshold is compared to the mean play audiometry threshold at age 4, across the 1-4 kHz range (second column), the 2-4 kHz range (third column) and with the mean audiometry threshold at age 8, across the 1-4 kHz range (fifth column) and the 2-4 kHz range (sixth column).*

*Table 1: Summary table of the difference of the mean between audiometry and ABR data, analyzed based on the shape of the audiogram.*

| ***Shape of audiogram*** | ***1-4 kHz avg age 4*** | ***2-4 kHz avg age 4*** | ***N*** | ***1-4 avg age 8*** | ***2-4 avg age 8*** | ***N*** |
| --- | --- | --- | --- | --- | --- | --- |
| ***Cookie-bite*** | *1,67* | *3,96* | *12* | *2,38* | *0,96* | *14* |
|  |  |  |  |  |  |  |
| ***Descending*** | *0,37* | *2,63* | *18* | *4,17* | *6,94* | *16* |
|  |  |  |  |  |  |  |
| ***Ascending*** | *5* | *1,5* | *5* | *7,5* | *2,5* | *2* |
|  |  |  |  |  |  |  |
| ***Flat*** | *4,45* | *4,17* | *3* | *8,75* | *8,75* | *4* |

*Examining the table reveals that the 1-4 kHz Pure-Tone Average (PTA) appears to be more aligned with audiograms exhibiting a descending shape. Meanwhile, the 2-4 kHz PTA appears to better correlate with ascending audiograms, while for flat and cookie-bite audiograms, no discernible distinction is evident between the 1-4 kHz and 2-4 kHz PTA.*

*In the case of 5 children, there was a disparity in the shape of their audiograms between ages 4 and 8, potentially attributable to a temporary additional conductive hearing loss impacting specific frequencies. However, we find the overall significance of this data to be inconclusive, primarily due to the limited number of children per category. Consequently, we have opted not to incorporate this analysis into our manuscript. We trust you concur with this decision. Furthermore, we have chosen to omit this from our discussion.*

Therefore, to be considered for publication, I recommend reevaluating and refocusing the scope of your work. It would be beneficial to concentrate on describing the results in detail, including the protocols used, and providing a more comprehensive description of the observed epidemiological findings.

**Answer:** *With the additional content and clarifications provided above, we trust that we have adequately addressed this aspect of your comment. We have incorporated more details into the description of our work, and in the methods section, we have explicitly outlined the protocols employed in our study.*

A detailed list of the suggestions by topic is given below:

Introduction:

L65: Describe the AABR acronym.

**Answer:** *Indeed, you are correct. I have now provided the complete expansion of the acronym.*

Methods:

It is very important to detail each of the protocols used, referencing them where appropriate.

ABR: please indicate equipment calibration, protocol for obtaining thresholds, derivation of electrodes (positive vertex?).

**Answer:** *We have supplemented details regarding the calibration of the ABR equipment and the specific placement of the vertex electrodes. You can find the protocol for obtaining thresholds outlined in lines 131-134 of the document.*

Visual reinforcement audiometry: Includes theoretical information on what the technique consists of and how it is recorded, but does not detail the protocol used.

**Answer:** *You are right. We happily included details about the measurement protocol we utilized.*

Ethics: I think it is important to mention the name of the ethics committee that approved the study protocol.

**Answer:** *We have incorporated this information into the designated section.*

Results:

I recommend a summary table with the most important data.

**Answer:** *We have attempted to add a summary table, as you suggested. We believe the table may provide a comprehensive overview of the data, so thank you for this suggestion. However, we also recognize that the table alone does not offer a representative portrayal of our data. Therefore, we have included the table along with a description emphasizing our belief that Figures 1-4 better capture the essence of our data.*

L245-246: It would be interesting to know the evolution of unilateral sensorineural hearing loss.

**Answer:** *Thank you for your interest, it is genuinely appreciated. We have now included information on the evolution of unilateral SNHL, following Figure 4 in the manuscript.*

L 250-252: This analysis can confuse the reader because if negative values are averaged with positive values, the result will tend to be 0. I recommend averaging with absolute values. This will better reflect what was observed in the Bland-Altman plots.

**Answer:** *We think you are absolutely right. This is the reason why we added the Bland-Altman plots for a fair depiction of our findings. Because of your comment, we now also changed the averaged values with averaged absolute values in these lines.*

Discussion:

I recommend strengthening the limitations and scope section of the study.

L290-293: As noted above, I feel that this statement does not reflect the actual scope of the work. I recommend it be reconsidered.

**Answer:** *Once again, we sincerely appreciate your insightful comments. We trust that, with the additional information provided in both this document and our manuscript, the statement now accurately captures the breadth of our work.*L256-357: Similar to the previous paragraph.

Reviewer #2: The article provides useful information for physicians treating children with hearing loss. The authors establish the relationships between ABR and audiometry in the first years of life, demonstrating the usefulness of click ABRs in predicting hearing loss PTAs. Only minor changes need be made.

line 65 and 72: put abbreviation for AABR in line 65, not 72.

**Answer:** *Thank you so much, I appreciate your attention to detail. The abbreviation is now correctly positioned.*

line 96: either 1 khz? fix this sentence

**Answer:** *The word “either” has been omitted.*

line 100: ends with ?

**Answer:** *We have verified, and changed “?” to “.”.*

line 104: you use PTA as the abbreviation for pure tone audiometry but also for pure tone average, please eliminate this abbreviation for pure tone audiometry, either change it or eliminate the need for abbreviation.

**Answer:** *We were unaware of this issue, and we appreciate your bringing it to our attention. Consequently, we have made the necessary adjustments, designating "PTA" as the abbreviation for Pure Tone Average and substituting "Pure Tone Audiometry" with the unabbreviated term "play audiometry."*

line 127 and 128: eliminate first sentence, not needed

line 129: doble .., eliminate

line 134: 31,1 should be 31.1

line 139: you already established abbreviation for SNHL, add it here

line 158: eliminate parenthesis for (play-)audiometry, not needed

line 174, 177: change aberrant

**Answer:** *Would “abnormal” be more suited?*

line 197: eliminate sentence about two sided test, not needed

line 295, change 5 to five

**Answer:** *We sincerely appreciate your valuable feedback. We have successfully incorporated all of your suggestions into the revised text.*
