# Supplementary material for: An eight-year follow-up on auditory outcomes after neonatal hearing screening
Source: PLoS One. 2024 Feb 28;19(2):e0297363. doi: 10.1371/journal.pone.0297363 (PMC10901348; doi:10.1371/journal.pone.0297363)
Supplement: Attachment — Submitted filename: Response to Reviewers.docx [file pone.0297363.s002.docx]

Dear Editor and Reviewers,

We extend our heartfelt appreciation to the reviewers and the editor for their invaluable insights and constructive comments. With the incorporation of the suggested modifications and additions, we hope to be reconsidered for publication.

In the section below, we offer response to the comments received.

With kind regards,

Jolien Kleinhuis

Also on behalf of the other authors:

Karin de Graaff, Paula van Dommelen, Irma van Straaten en Ruben Benard

**Additional Editor Comments:** Reviewer 1 has raised concerns regarding the ABR sample size (n=30) at 8 years old. It is crucial to address this limitation explicitly in the discussion section. Please add a caveat.

**Answer:** We have carefully considered the concerns about the ABR sample size at 8 years old and we understand the concerns of Reviewer 1. Therefore we made two major changes in the manuscript.

First of all, we reformulated the manuscript towards a more descriptive approach, as Reviewer 1 suggested. We changed the objective in the abstract (line 20-21), in the introduction (line 81-88), in the method (line 102-104) and in the discussion (line 291-294).

Secondly, according to the suggestion of the Editor, we added a caveat to our discussion, aiming to explicitly address this limitation (line 349-352). These changes towards the descriptive approach resulted in a reformulation of the title (line 1) and the conclusion (line 360-363).

Finally, we made minor adjustments in two sentences to enhance readability (line 157 and 313).

Overall, we believe these refinements contribute positively to the overall strength of this manuscript and we thank the Editor and Reviewer 1 for bringing this to our attention.

**Comments to the Author**

Reviewer #1: Dear Authors,

I would like to express my appreciation for your response to my previous comments on the revised version of your manuscript.

After careful consideration of the revisions made, particularly those addressing minor concerns, I find that there are still aspects that require attention, particularly with regard to the primary objectives related to the predictive value of neonatal click ABR in healthy children with respect to their perceptual auditory thresholds throughout life up to the age of 8 years.

As mentioned in my original report, the number of subjects at 8 years of age (n=30) appears to be low to fully address the objectives outlined in the article. Furthermore, the predictive value of ABR with behavioural thresholds is well established. Therefore, if the incidence of fluctuating hearing loss is low or excluded from the analysis, the predictive value will naturally remain high as long as the hearing level remains stable, regardless of age.

Under these circumstances, I regret to inform you that I am unable to recommend your manuscript for publication. However, as I mentioned in the initial report, I appreciate the reported time period of the extended cohort and the technical quality of the reported measurements. Once again, I recommend that the manuscript be reformulated towards a more descriptive approach. In such a case, it is the responsibility of the editorial team to determine whether the relevance and impact of the paper meet the requirements of PLOS ONE.

Best Regards

**Answer:** In the first place, we want to thank you for the effort you have made to bring your concerns about the article to our attention. We agree with you; our initial objective of the manuscript focused on the predictive value of the click ABR, but the limited sample size imposes constraints on the generalizability of our findings.

Therefore, following your suggestions, we have made the document more descriptive. As mentioned in the response to the Editor as well, we changed the objective in the abstract (line 20-21), in the introduction (line 81-88), in the method (line 102-104) and in the discussion (line 291-294). Furthermore, we added a caveat to our discussion, aiming to explicitly address this limitation (line 349-352). These changes resulted in a reformulation of the title (line 1) and the conclusion (line 360-363).

Furthermore, thanks to your comments about the number of subjects, we made changes in description of the sample size (line 29-31 and 313) to clarify the number of children successfully finishing the 8-year follow-up.

Your input has been of great value, and we hope these changes improve alignment with your expectations.
